# Faecal calprotectin level for assessing endoscopic activity and predicting future clinical course in patients with moderately active ulcerative colitis undergoing granulomonocytapheresis: a prospective cohort study

**DOI:** 10.1186/s12876-018-0853-4

**Published:** 2018-08-01

**Authors:** Takahiro Shimoyama, Takayuki Yamamoto, Satoru Umegae, Koichi Matsumoto

**Affiliations:** Inflammatory Bowel Disease Centre, Yokkaichi Hazu Medical Centre, 10-8 Hazuyamacho, Yokkaichi, 510-0016 Japan

**Keywords:** Ulcerative colitis, Endoscopic activity, Faecal calprotectin, Granulomonocytapheresis, Predicting relapse

## Abstract

**Background:**

Calprotectin is a stable neutrophil protein, which can be measured in faecal samples. The faecal level of calprotectin increases during disease activity in ulcerative colitis (UC). Nonetheless, the relevance of faecal calprotectin (FC) measurement during granulomonocytapheresis (GMA) for UC has not yet been fully evaluated. This prospective study was to investigate the value of FC for assessing disease activity and predicting clinical course in UC patients undergoing GMA therapy.

**Methods:**

One hundred and eighty-four patients with moderately active UC with endoscopic activity (Mayo endoscopic subscore [MES] = 2 or 3) received Adacolumn GMA therapy (10 apheresis sessions over consecutive 5 weeks). Patients who achieved clinical remission were subsequently given maintenance medications for 12 months. FC levels were measured at entry and after treatment.

**Results:**

After GMA, 80 of the 184 patients (43%) achieved clinical remission, and 51 (28%) achieved mucosal healing (MH; MES = 0 or 1). The median FC level significantly decreased in patients who achieved MH (*P* = 0.02), but not in those without MH. Thirty-four patients (43%) relapsed during the 12-month follow-up. The median FC level at the end of GMA therapy was significantly higher in patients who subsequently relapsed than in those who maintained remission (149.5 vs 45.5 μg/g, *P* < 0.001). A cut off value of 114 μg/g had a sensitivity of 76% and a specificity of 85% to predict future relapse.

**Conclusions:**

Our findings indicate that FC is a relevant biomarker, which is convenient to measure for assessing endoscopic activity and predicting relapse in patients who achieve remission following a course of GMA therapy.

## Background

In Japan, since April 2000, granulomonocytapheresis (GMA) with an Adacolumn has been approved by the Japan Ministry of Health as one treatment option for patients with active ulcerative colitis (UC) [1–5]. The mechanism of actions of GMA includes selective depletion of elevated and activated myeloid lineage leucocytes (granulocytes and monocytes/macrophages), which are perceived to be part of the immune disorder in patients with inflammatory bowel diseases (IBD) [1–5]. One unique feature of GMA is that it spares red blood cells [1]. Previous studies have reported that GMA was effective in patients with mild to moderately active UC and had a favourable safety profile [6–16]. Currently, GMA is widely used in Japan and is available in the European Union countries.

Calprotectin is a calcium-binding protein, a member of the S100 family, abundant in the cytoplasm of neutrophils [17, 18]. During active IBD, neutrophils infiltrate the intestinal mucosa and an increase in faecal calprotectin (FC) level is the consequence of neutrophil degranulation and tissue damage within the intestinal mucosa. Calprotectin is found to be stable in faecal samples for up to 7 days at room temperature [19]. FC testing is now widely used as a non-invasive surrogate marker of intestinal inflammation in patients with IBD [20–22]. The clinical application of FC includes differentiation of irritable bowel syndrome from classical IBD, assessment of disease activity, response to medications, and prediction of clinical course [23–25].

Previous studies evaluating the relevance of FC in IBD, targeted patients who were treated with pharmacologicals, while the diagnostic value of FC during GMA therapy has not been reported. We thought that FC measurement might be a relevant undertaking in GMA settings. Accordingly, we have focused on FC testing for assessing the efficacy of GMA and for predicting relapse in patients who achieved remission following a course of GMA therapy.

## Methods

### Study plan

**Fig. 1:**
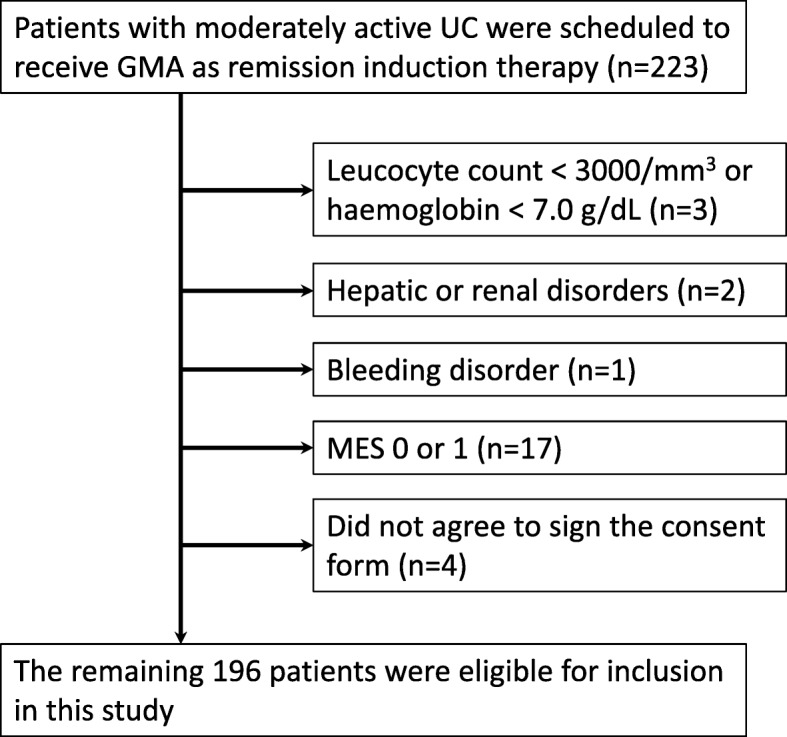
A flow diagram summarising patient selection and exclusion. Twenty-seven patients were excluded, and the remaining 196 patients were included in this study. UC, ulcerative colitis; GMA, granulomonocytapheresis; MES, Mayo endoscopic subscore

This was a prospective cohort study undertaken at the Yokkaichi Hazu Medical Centre. Inclusion criteria were: 1) endoscopic and histologic diagnosis of UC; 2) moderately active UC with Mayo score [26] 6–9; 3) Mayo endoscopic subscore (MES) of 2 (moderate) or 3 (severe); 4) active disease despite receiving one or more of the following medications, 5-aminosalicylic acid (5-ASA) preparations (sulphasalazine or mesalazine), corticosteroid, thiopurines (azathioprine or 6-mercaptopurine), tacrolimus, cyclosporine, biologics (infliximab, adalimumab, or golimumab); 5) agreed to undergo endoscopic examinations at entry and after GMA therapy; 6) agreed to provide a stool sample for the measurement of FC. Exclusion criteria were: 1) leucocyte count < 3000/mm^3^ or haemoglobin < 7.0 g/dL; 2) malignancy; 3) serious concomitant cerebral, pulmonary, cardiac, hepatic or renal disorders; 4) bleeding disorder, or a history of hypersensitivity reaction to an anticoagulant; 5) toxic megacolon, fulminating colitis, peritonitis or sepsis. A total of 223 patients with moderately active UC scheduled for GMA therapy were screened for eligibility. A flow diagram summarising patient selection and exclusion is presented in Fig. 1. A total of 196 patients were eligible for inclusion.

### GMA therapy

Each patient was scheduled to receive a total of 10 GMA sessions with the Adacolumn (JIMRO, Takasaki, Japan) over 5 consecutive weeks. One GMA session was about 90 min at 30 mL/minute. The Adacolumn is an adsorptive carrier based granulocyte and monocyte apheresis device with a volume of 335 mL, filled with about 220 g cellulose acetate beads of 2 mm diameter as the column adsorptive leucocytapheresis carriers [1, 27]. In patients who were receiving corticosteroid at entry, the dose of the steroid was to be tapered or discontinued in line with clinical improvement. Patients receiving 5-ASA, thiopurine or biologic at entry could continue these medications at the same dosage.

### Assessment of clinical efficacy

Clinical assessment was regularly undertaken during GMA treatment course. The clinical symptom score was defined as the sum of ‘stool frequency’ score (0–3) and ‘rectal bleeding’ score (0–3) in the Mayo scoring system [26]. Two previous studies [28, 29] have found that this 6-point score correlates very well with the partial (stool frequency, rectal bleeding, and the physician’s global assessment) and full Mayo scores (stool frequency, rectal bleeding, endoscopic findings, and a physician’s global assessment). The 6-point score was compared at entry (within 1 week before the first GMA session) and after treatment (within 2 weeks after the last GMA session). Clinical remission was defined as a score of 0 after GMA treatment. Clinical improvement was defined as a decrease in the clinical symptom scores by at least 1 point.

### Endoscopic assessment

At entry, complete endoscopic evaluation, up to the caecum was made. After treatment, mainly, the most affected segment at entry was observed. Then, the endoscopic severity of the most inflamed segment was compared after GMA therapy relative to baseline. Mucosal healing (MH) was defined as an MES of 0 or 1 after treatment. Endoscopic severity was assessed by physicians who were blinded to the clinical data and the results of the FC testing.

### Measurement of faecal calprotectin

FC measurement was done at entry, after treatment and also when a patient relapsed after achieving clinical remission with GMA. Patients were advised to collect a stool sample in the early morning within 5 days before their clinic visit and store at room temperature. From the clinic, faecal samples were sent to laboratory, and FC was measured by a quantitative enzyme immunoassay (Human Calprotectin Enzyme-linked immunosorbent assay Kit, Cell Sciences Inc., Massachusetts, USA). Laboratory personnel were blinded to the clinical data.

### Maintenance medication

After GMA therapy, all patients except those who required colectomy were given maintenance medication and were regularly followed for > 12 months. Patients who did not respond to GMA were treated with an increased dose of corticosteroid, immunosuppressant, biologic, or underwent colectomy. For patients who achieved clinical remission with GMA, the dose of corticosteroid was to be tapered or discontinued if they were still on steroid at the end of GMA therapy. Medication with 5-ASA, immunosuppressant or biologic was continued as maintenance therapy, and no other medication was added unless a relapse occurred. Relapse was defined as worsening of the clinical symptom score with the MES of 2 or 3.

### Statistics

Comparisons of frequencies were done by using the *χ*^2^ test with Yates’ correction. Differences between median values were compared with the Mann-Whitney U test or the Kruskal-Wallis test if more than two groups were to be compared. The change in median values with time was analysed by applying the Wilcoxon signed rank test. The cumulative relapse rate after GMA therapy was calculated by the Kaplan-Meier estimator graphs, and was compared between the groups by using the log-rank test. *P* < 0.05 was considered statistically significant. To determine an optimal cut off value for the prediction of relapse, a receiver operating characteristic curve was constructed.

## Results

### The overall efficacy outcomes

**Fig. 2:**
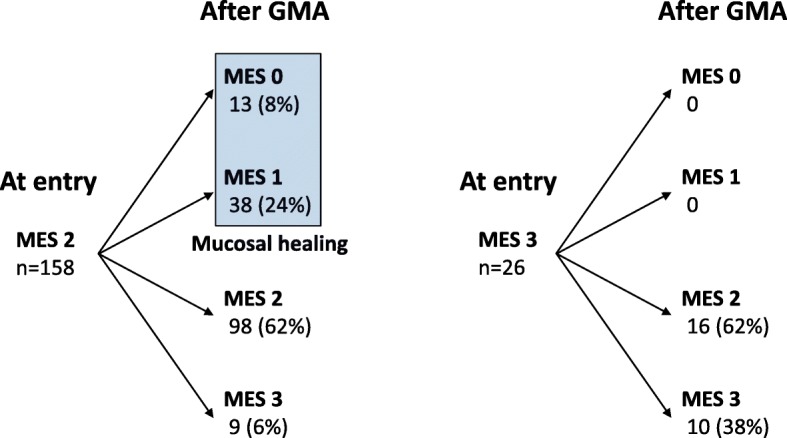
The change of endoscopic severity during the GMA treatment course. Mucosal healing was observed in 51 of the 158 patients (32%) with the Mayo endoscopic subscore (MES) 2 at entry vs none of the 26 patients with the MES 3 (*P* = 0.002)

GMA therapy was initiated in 196 patients who met the inclusion criteria, but another 12 patients were withdrawn during the study period for the following reasons. Two patients required emergency colectomy, 3 were intolerant to GMA due to adverse events (headache or nausea), 3 could not undergo endoscopic examination after the treatment, 2 could not provide faecal samples, and 2 were lost to follow-up. Therefore, 184 of the 196 patients were eligible for assessing the GMA efficacy outcomes. The baseline characteristics of the patients are presented in Table 1. The median (interquartile rage [IQR]) of the clinical symptom score significantly decreased during GMA therapy, from 4 (4–5) at entry to 2 (0–5) after treatment (*P* < 0.001). An 80 (43%) of the 184 patients achieved clinical remission, and 40 (22%) showed clinical improvement without achieving complete remission. At entry, the MES of the most severely affected intestinal segment was 2 in 158 patients (86%) and 3 in 26 patients (14%) (Fig. 2). Clinical remission was achieved in 72 of the 158 patients (46%) with an MES of 2 at entry vs 8 of 26 (31%) with an MES of 3 (*P* = 0.23).

**Table 1:** Baseline characteristics of the 184 patients

Median (IQR) age at entry	38.5 (29–50) years
Male: female (n)	99: 85
Median (IQR) duration of UC before entry	33 (22–45) months
Extent of UC (n)	
Proctosigmoiditis	35 (19%)
Left-sided colitis	119 (65%)
Extensive colitis^a^	30 (16%)
Extraintestinal manifestations (n)	
Arthritis	8 (4%)
Erythema nodosum/pyoderma gangrenosum	2 (1%)
Induction therapy for the current exacerbation (n)	
Mesalazine (Pentasa 4 g/day)	174 (95%)
Corticosteroids (Prednisolone 30–60 mg/day)	149 (81%)
Thiopurines (Azathioprine 50–100 mg/day)	39 (21%)
Calcineurin inhibitors	0
Biologics	0
Mayo endoscopic subscore (MES) at entry	
MES 2	158 (86%)
MES 3	26 (14%)

*IQR* interquartile rage, *UC* ulcerative colitis

^a^Involvement extends proximal to the splenic flexure

The change in endoscopic severity during the GMA treatment course is presented in Fig. 2. Overall, MH was observed in 51 of the 184 patients (28%). When sub-grouped, MH was achieved in 51 of 158 patients (32%) with MES 2 at entry, which was significantly higher than none of the 26 with MES 3 (*P* = 0.002). Further, MH was achieved in 45 of 80 patients (56%) who achieved clinical remission vs 6 of 104 (6%) without clinical remission (*P* < 0.001). Age, gender, duration of UC before entry, extent of UC, or extra-intestinal manifestations did not affect the endoscopic efficacy of GMA.

### Faecal calprotectin vs disease activity

**Fig. 3:**
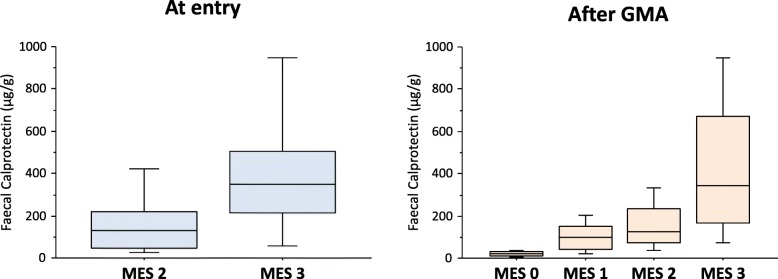
Endoscopic severity (MES) both at entry and after GMA treatment showed significant positive relationship with the levels of faecal calprotectin. At entry, the median faecal calprotectin level was significantly higher in patients with MES 3 than in those with MES 2 (*P* < 0.001 by the Mann-Whitney U test). After GMA treatment, the median faecal calprotectin level was significantly higher in patients with higher MES (*P* < 0.001 by the Kruskal-Wallis test)

The overall, median (IQR) of FC level significantly decreased from 140 (52–317) μg/g at entry to 121 (47–219) μg/g after treatment (*P* = 0.03). In patients who achieved clinical remission, the median (IQR) of FC was 99 (41–206) μg/g at entry vs 75 (30–160) μg/g after treatment (not significantly different). In patients who did not achieve clinical remission, FC was 181 (77–367) μg/g at entry vs 142 (81–259) μg/g after treatment (not significantly different). Endoscopic severity (MES) before or after treatment showed significant positive relationship with FC level (Fig. 3). Further, in patients who achieved MH, the median (IQR) of FC decreased from 59.5 (31.5–193.5) μg/g at entry to 52 (24–128) μg/g after treatment (*P* = 0.02), while in those without MH, it did not change significantly, 153 (69–340) μg/g at entry vs 142 (77–263) μg/g after treatment.

### The outcomes of post GMA maintenance therapy

**Fig. 4:**
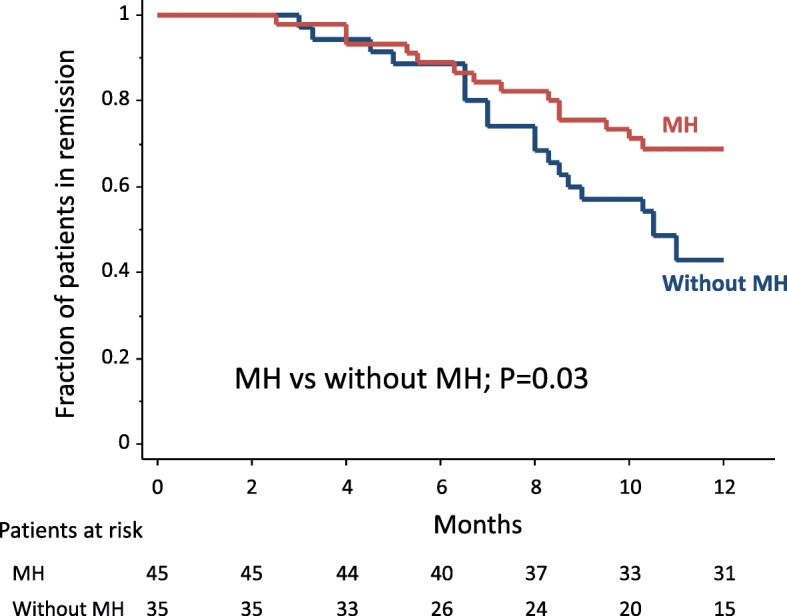
The cumulative relapse rate was significantly higher in patients without mucosal healing (MH) compared with those with MH

The 80 patients who achieved clinical remission received maintenance mesalazine (*n* = 62) or azathioprine (*n* = 18). Thirty-four (43%) patients relapsed during the 12-month follow-up. Relapse rates were not significantly different between the 2 medications, 44% vs 39%, respectively. Relapse was observed in 14 of 45 patients (31%) who achieved MH vs 20 of 35 (57%) without MH (*P* = 0.04). Similarly, the cumulative relapse rate was significantly higher in patients without MH compared with those with MH (Fig. 4). In contrast, the following measurements did not affect the relapse rate, age, gender, duration and the extent of UC, or extra-intestinal manifestations. In the 34 patients who relapsed, the median (IQR) FC had significantly increased from 149.5 (96–211) μg/g at the end of GMA therapy to 195.5 (119.5–250) μg/g at the time of relapse (*P* = 0.02). These 34 patients were treated with corticosteroid, immunosuppressant, biologic, or additional GMA sessions, and 30 of these 34 patients (88%) achieved clinical remission again. In the 30 responders, the median (IQR) FC level significantly decreased from 203 (114–273.5) μg/g to 101.5 (58.5–184) μg/g after treatment (*P* = 0.02). The remaining 4 patients with unremitting UC underwent colectomy.

### FC level and future relapse

**Fig. 5:**
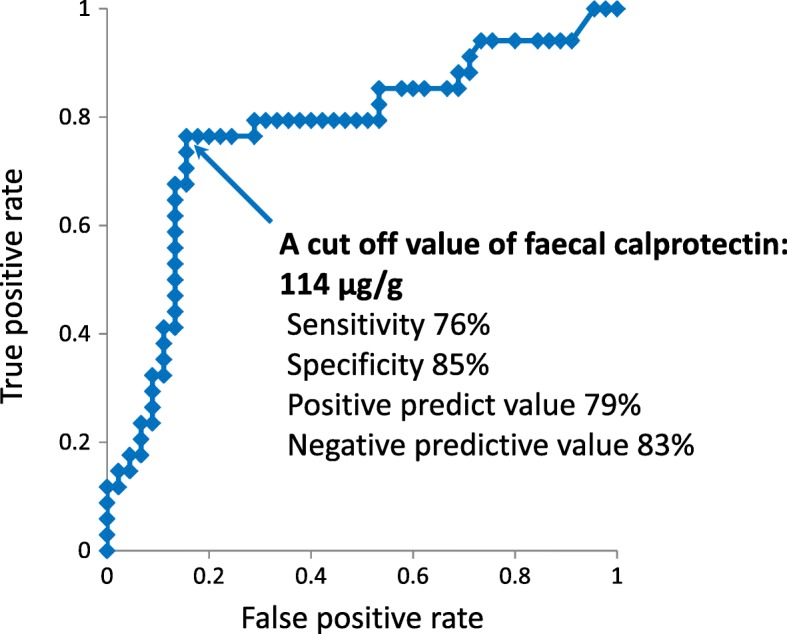
The receiver operating characteristic curve showed that the optimal cut off level of faecal calprotectin for the prediction of clinical relapse was 114 μg/g

**Fig. 6:**
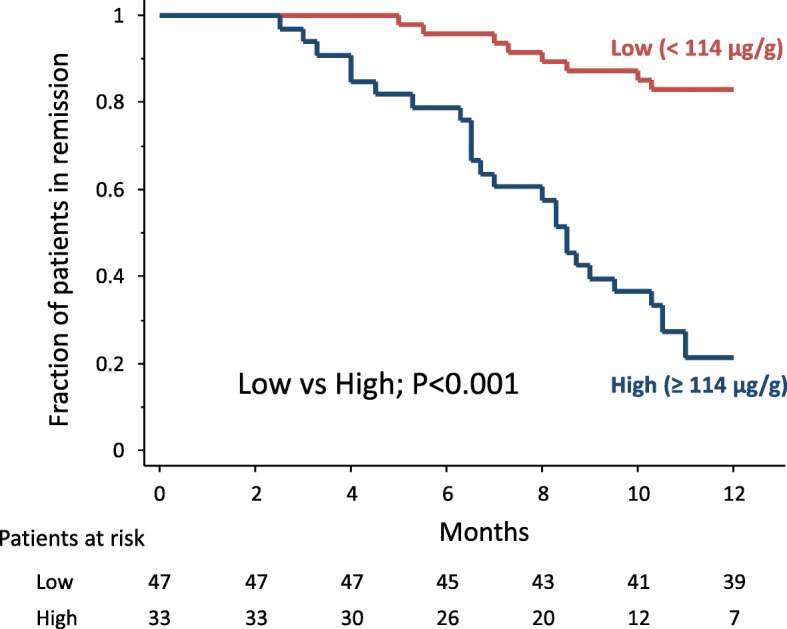
The cumulative relapse rate was significantly higher in patients with elevated FC level (≥114 μg/g) compared with those with low FC level (< 114 μg/g)

The median (IQR) FC level at the end of GMA therapy was significantly higher in patients with relapse than in those without relapse, 149.5 (96–211) μg/g vs 45.5 (23–99) μg/g (*P* < 0.001). A cut off value of 114 μg/g FC had a sensitivity of 76% (95% confidence interval [CI]: 62–91%), a specificity of 85% (95% CI: 74–95%), a positive predict value (PPV) of 79% (95% CI: 65–93%), and a negative predictive value (NPV) of 83% (95% CI: 72–94%) to predict future relapse (Fig. 5). Relapse was observed in 26 of 33 patients (79%) with elevated FC (≥114 μg/g), but in 8 (17%) of 47 patients with low FC (< 114 μg/g) (*P* < 0.001). Similarly, the cumulative relapse rate was significantly higher in patients with elevated FC (≥114 μg/g) compared with those with low FC (< 114 μg/g) (Fig. 6).

## Discussion

To our knowledge, this is the first study, which has fully evaluated the clinical relevance of FC measurement in UC patients under the Adacolumn GMA therapy. Our study has several strengths. First, it was prospectively designed and conducted with a relatively large number of patients. We included patients with homogeneous background regarding clinical disease activity at baseline and induction interventions for the current UC exacerbation. Second, all patients underwent endoscopic assessments before and after GMA treatment course, which was expected to allow assessing the true endoscopic efficacy of GMA. Third, all patients received the same treatment regimen during GMA therapy.

Regarding the efficacy outcomes during GMA therapy, 43% of patients achieved clinical remission, and 28% achieved MH. MH was more frequently observed in patients with moderate endoscopic activity at entry vs those with severe endoscopic activity (32% vs 0%). After achieving clinical remission, the patients were treated with maintenance mesalazine with or without azathioprine. However, concomitant azathioprine did not significantly affect the remission rate. In this study, azathioprine therapy was not started during GMA therapy. It was continued during the study period in patients who did not respond to it before entry. Therefore, we presume that the efficacy of azathioprine as maintenance therapy was also limited in such patients. The relapse rate was significantly higher in patients who did not achieve MH during GMA therapy than in those who achieved MH (57% vs 31%). The following demographic factors did not affect the relapse rate, age, gender, duration and extent of UC, or extra-intestinal manifestations.

However, endoscopic features appeared to be key in assessing and predicting the efficacy of GMA therapy. Further, endoscopic severity of mucosal inflammation showed significant correlation with FC levels, both before and after GMA therapy. The median FC level significantly decreased in patients who achieved MH, but did not change significantly in those without MH. However, fast and sharp reduction in FC levels during treatment with biologics was not observed in the present study because GMA was not indicated for those with severe disease with markedly elevated FC levels.

In a smaller study, Hanai, et al. [30] reported that FC level showed a significant correlation with endoscopic severity in UC patients, and significantly decreased during GMA therapy. A significant correlation with endoscopic activity demonstrates that FC represents a clinically relevant biomarker for monitoring endoscopic inflammation in UC patients during GMA therapy. A close correlation between endoscopic severity and FC levels has also been reported in patients with UC during drug therapy [31–34]. FC is derived mainly from activated neutrophils in the inflamed intestinal mucosa [17–19].

The median FC level at entry was lower in patients who achieved clinical remission than in those who did not (99 vs 181 μg/g). We speculate on the reasons behind these findings. In patients without subsequent clinical remission, the proportion of cases with MES 3 (vs MES 2) was higher compared with those with subsequent clinical remission (17% vs 10%). FC level was markedly elevated in patients with MES 3 (vs MES 2) as shown in Fig. 3. This is why FC level was lower in those with subsequent clinical remission.

The relapse rate after GMA was significantly higher in patients who did not achieve MH during GMA therapy than those who achieved MH. MH is now a known predictor of clinical relapse during maintenance drug therapy following GMA. Likewise, patients with high FC levels were at a higher risk for clinical relapse as compared with those with low FC. For predicting clinical relapse, the accuracy of FC (cut off value = 114 μg/g) appeared to be appropriate (sensitivity = 76%, specificity = 85%), while a high NPV (83%) indicated that unnecessary endoscopic examination can be avoided in patients with low FC. In contrast, patients with high FC at the end of GMA therapy should receive more intensive medical therapy and be diligently monitored to avoid an early relapse. Accordingly, FC monitoring during remission should be a relevant approach for predicting relapse. Similar findings on the use of FC were also observed in studies that had targeted patients who achieved remission during drug therapy [20, 32, 35–37]. We believe that monitoring FC can detect an ongoing, low level background inflammation in the intestinal mucosa, which may grow and lead to a clinical relapse.

Our study has certain limitations. First, FC was measured at baseline and at the end of GMA treatment course, it was not consecutively measured during maintenance drug therapy after GMA. Additional clinical research is required to ascertain if serial monitoring of FC leads to an improved predictability for subsequent relapse after GMA-induced remission. Second, in this investigation, patients who did not respond to a calcineurin inhibitor (tacrolimus) or biologic at baseline were not included because the non-responders had severe active disease. The value of FC in more severe active UC should be assessed in the future.

In our study, FC levels are lower compared with other studies with active IBD [20, 31, 33, 34]. This discrepancy could be attributed to the different assays used for the determination of FC levels. It was reported that large quantitative differences were observed between the different FC assays [38]. This variation makes it impossible to use methods interchangeably, and enforces the urgent need for standardisation in measurement procedures.

## Conclusions

We found that FC, which is convenient to measure in patients with IBD could become a validated biomarker for the assessment of endoscopic disease activity in UC patients undergoing GMA therapy. Further, FC at the end of GMA treatment course appeared to be a relevant biomarker for the prediction of clinical relapse in patients who had achieved remission. Further, FC measurement is a relatively low-cost approach to monitor intestinal inflammation, and should spare patients from invasive and time-consuming endoscopic procedures. Our FC levels appeared to be lower compared with previous studies in which different FC assays were used. Additional, well designed, prospective, controlled trials with standardisation in FC measurements should strengthen our findings. Future studies should investigate whether early medical intervention can prevent subsequent relapse in patients with elevated FC.

## Abbreviations

CI: Confidence interval
FC: Faecal calprotectin
GMA: Granulomonocytapheresis
IBD: Inflammatory bowel disease
IQR: Interquartile rage
MES: Mayo endoscopic subscore
NPV: Negative predictive value
PPV: Positive predict value
UC: Ulcerative colitis (UC)
